# Crystal structure, Hirshfeld surface, DFT, mol­ecular docking of 1-[(6-*tert*-butyl-2-oxo-2*H*-chromen-4-yl)meth­yl]-4,4-di­methyl­piperidine-2,6-dione and cytotoxic effects on breast cancer (MDA-MB 231), human alveolar basal epithelial (A549) cell lines

**DOI:** 10.1107/S2056989025001550

**Published:** 2025-02-25

**Authors:** M. Sunitha Kumari, M. Harish Kumar, D. V. Deevith, H. C. Devarajegowda, B. S. Palakshamurthy

**Affiliations:** a Department of Physics Yuvaraja's College University of Mysore,Mysore 570005 Karnataka India; b Dr. B. R. Ambedkar Medical College, Gandhi nagar, Kadugondanahalli, Bangalore-560045, Karnataka, India; c Department of PG Studies and Research in Physics Albert Einstein Block UCS Tumkur University, Tumkur Karnataka-572103 India; Institute of Chemistry, Chinese Academy of Sciences

**Keywords:** crystal structure, 2-oxo-2*H*-chromene, DFT, biological activity, Hirshfeld surface

## Abstract

The title compound, C_21_H_25_NO_4_, was synthesized by S_N_2 reaction of bromo­methyl coumarin with 4,4-di­methyl­piperidine-2,6-dione. The mol­ecule crystalizes in the monoclinic system with space group *C*2/*c*. The coumarin unit is almost planar with a dihedral angle between the aromatic rings of 0.81 (2)° and an r.m.s deviation of 0.042 Å. The piperidine ring adopts a chair conformation with the two methyl groups, one methyl group occupying an axial position and the other an equatorial position, exhibiting maximum stability. In the crystal, C—H⋯O inter­actions lead to the formation of head-to-head dimers with an *R*_2_^2^(8)graph-set motif and *R*_2_^1^(9) and *R*_2_^2^(10) ring motifs along [001] and [100]. π–π inter­actions [centroid–centroid distances = 3.885 (2) and 3.744 (2) Å] are also observed. A Hirshfeld surface analysis was carried out, with the two-dimensional fingerprint plots indicating that the major contributions to the crystal packing are from H⋯H(57%), O⋯H(29.3%) and C⋯H(8.1%) inter­actions. The energy framework calculations reveal that dispersion energy (*E*_dis_= −267.7 kJ mol^−1^) dominates the other energies. The mol­ecular structure was optimized by density functional theory calculations using the B3LYP/6–311+G(d,p) basis set. The HOMO and LOMO orbitals were generated to determine the energy gap, which is 4.245 eV. Mol­ecular docking studies were carried out for the title mol­ecule as ligand and a protein as receptor giving binding affinities of −9.5 kcal mol^−1^ for PDB ID: 5HG8 and −8.2 kcal mol^−1^ for PDB ID:6 NLV. The compound was further subjected to biological studies against human cancer cell lines, namely cryopreserved triple negative human breast adenocarcinoma cells (MDA-MB-231cells) and adenocarcinomic human alveolar basal epithelial cells (A549 cells) giving IC_50_values of 11.57 and 9.34 µ*M*, respectively. The cytotoxicity results showed a good safety profile against HEK293 cell lines.

## Abstract 2

The title compound was synthesized by S_N_2 reaction of bromo­methyl coumarin with 4,4-di­methyl­piperidine-2,6-dione. Its crystal structure was determined and a Hirshfeld surface analysis was performed along with DFT, mol­ecular docking and biological activity studies.

## 1. Chemical context

Coumarin and its derivatives are considered to be significant heterocyclic compounds. These compounds possess structural features that offer several types of biological and pharmaceutical effects such as vasodilation, nitrate-coumarin derivatives in particular being considered to be potent mol­ecules for the inhibition of the vasodilator effect (Matos *et al.*, 2022 ▸). The combination of coumarin and 7-hy­droxy­coumarin plays significant role in the inhibition of the growth of a number of malignant cells of murine and human origin, and hence they are considered to be good anti-tumor, immunomodulation agents (Stefanova *et al.*, 2007 ▸). Pyrimidino-coumarin derivatives have been found to exhibit platelet anti-aggregatory activity as well as being anti­thrombotic agents, which are being developed as commercial drug mol­ecules (Ramsis *et al.*, 2023 ▸). Much research effort has been made to derive coumarin from herbal products, naturally derived coumarin being found to exhibit neuro-protective (Wang *et al.*, 2012 ▸) and anti-ageing properties, which makes coumarin widely used in the cosmetic industry (Costa *et al.*, 2022 ▸). Coumarin is available in several chemical subgroups that possess significant pharmacological and toxicological properties and plays an important role with regard to cardiovascular health in humans and wound healing (Najmanova *et al.*, 2015 ▸; Afshar *et al.*, 2020 ▸). Keeping all these factors in mind, our team synthesized the title 6-*tert*-butyl-2*H*-chromen-substituted mol­ecule and studied its crystal structure, along with its cytotoxic effects on breast cancer (MDA-MB 231) and human alveolar basal epithelial (A549) cell lines and performed mol­ecular docking studies, which are reported herein.
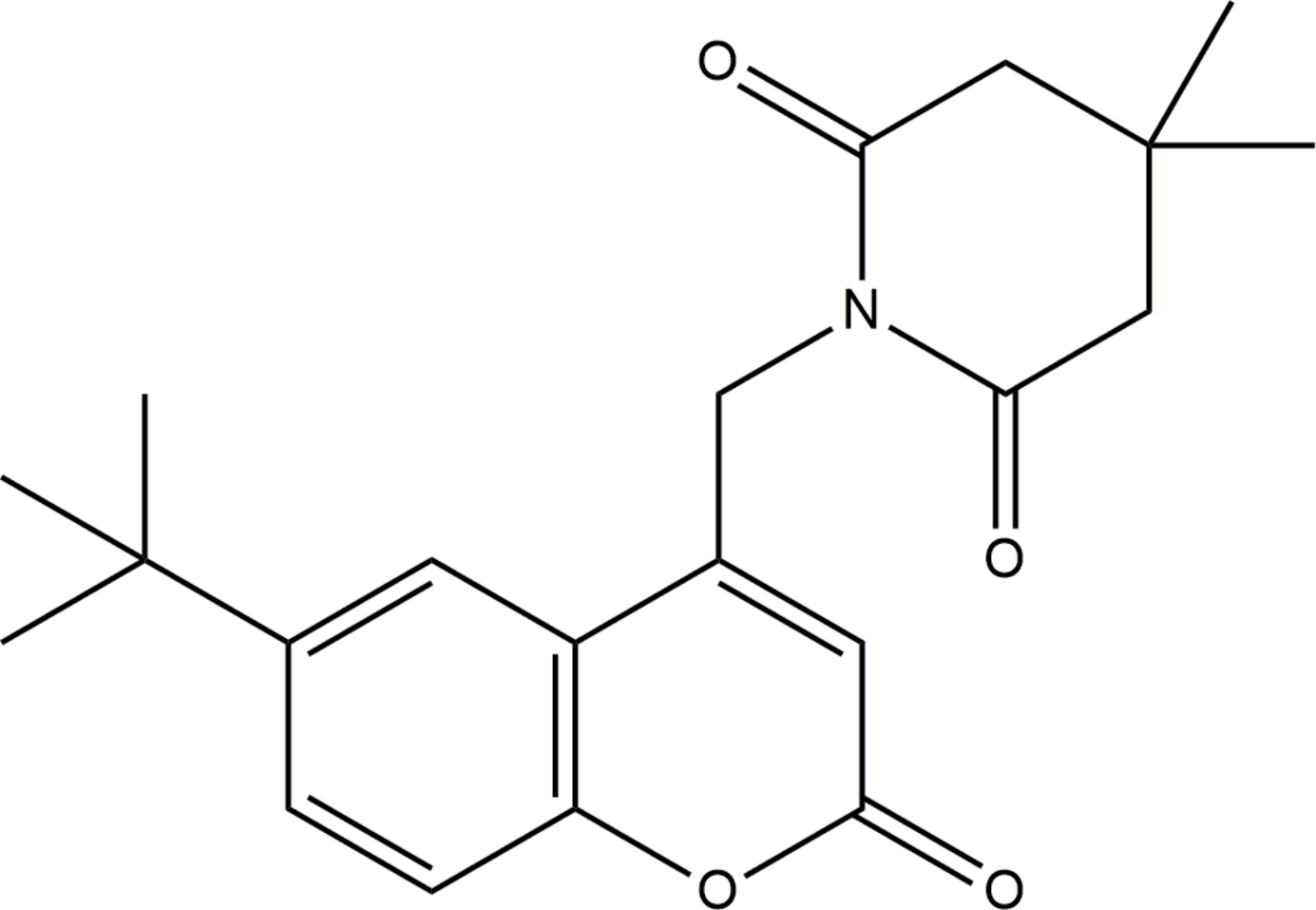


## 2. Structural commentary

**Figure 1:**
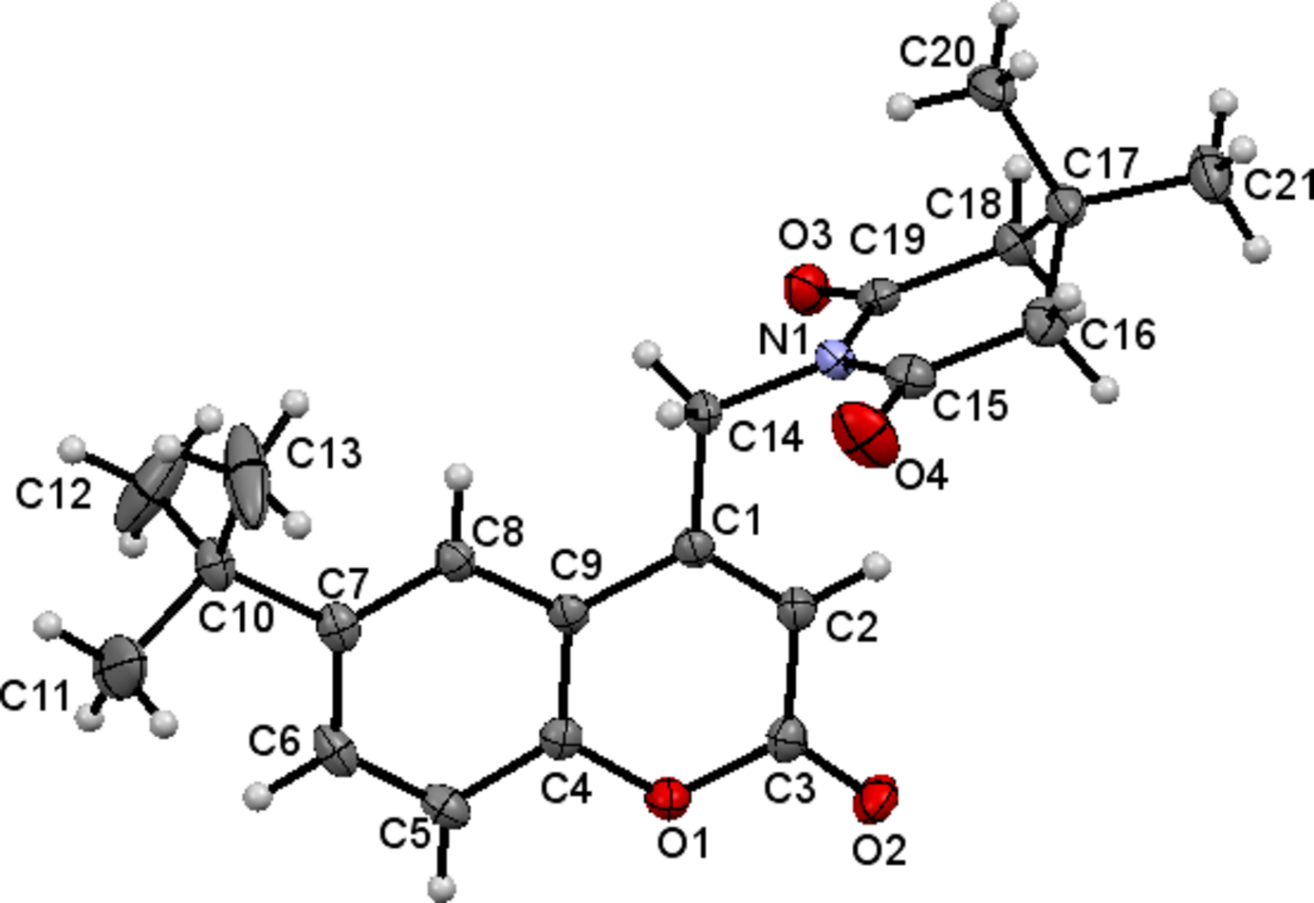
The mol­ecular structure of (I) with displacement ellipsoids drawn at the 50% probability level.

The mol­ecular structure of the title compound, (I), is shown in Fig. 1 ▸. The coumarin (ten-membered ring system) is almost planar with a dihedral angle 0.81 (2)° between the aromatic rings and an r.m.s deviation of 0.042 Å. The piperidine ring mol­ecule adopts a half-chair conformation. The six-membered N1/C15–C19 ring has a total puckering amplitude (*Q*) of 0.4646 (17)Å and exhibits a half-chair conformation. The pseudo rotation (θ) and the relative phase (φ) angles are 53.8 (2) and 181.3 (3)°, respectively. The two methyl groups are attached to atom C17, one occupying an axial position and the other an equatorial position. The dihedral angle between the mean planes of the coumarin ring system and the piperidine ring is 83.07 (6)°.

## 3. Supra­molecular features

**Figure 2:**
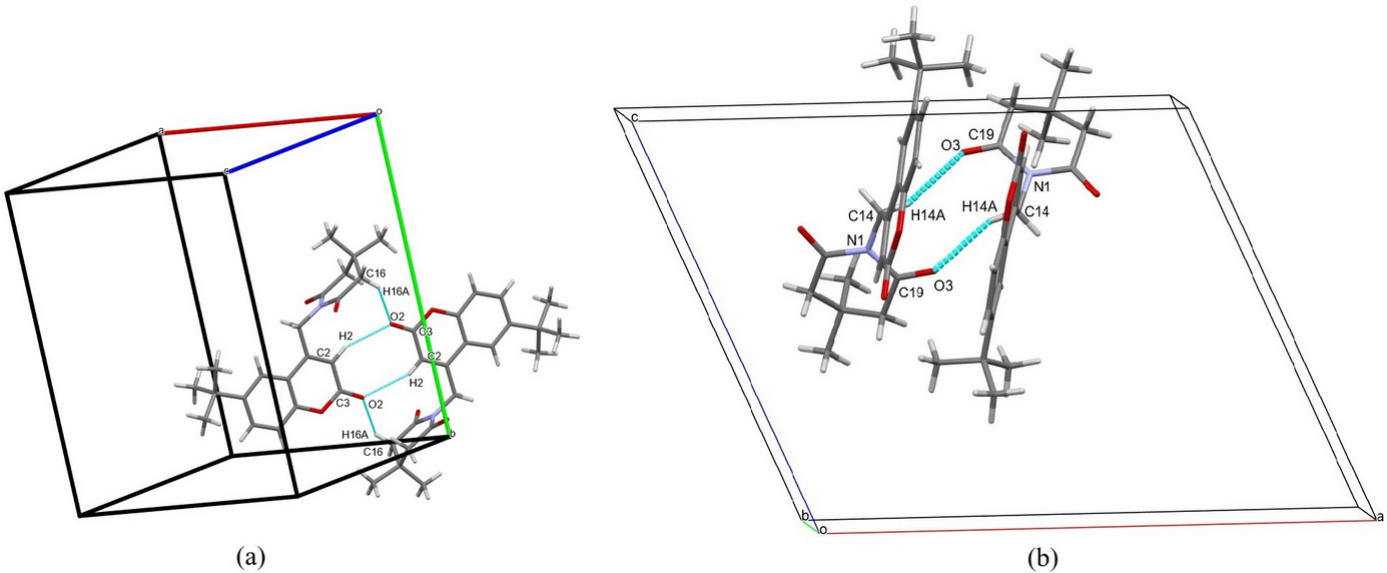
The mol­ecular packing of (I) with C—H⋯O inter­actions depicted by dashed lines, (*a*) showing the 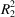
(8), 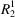
(9) synthon and (*b*) showing the 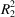
(10) synthon.

**Figure 3:**
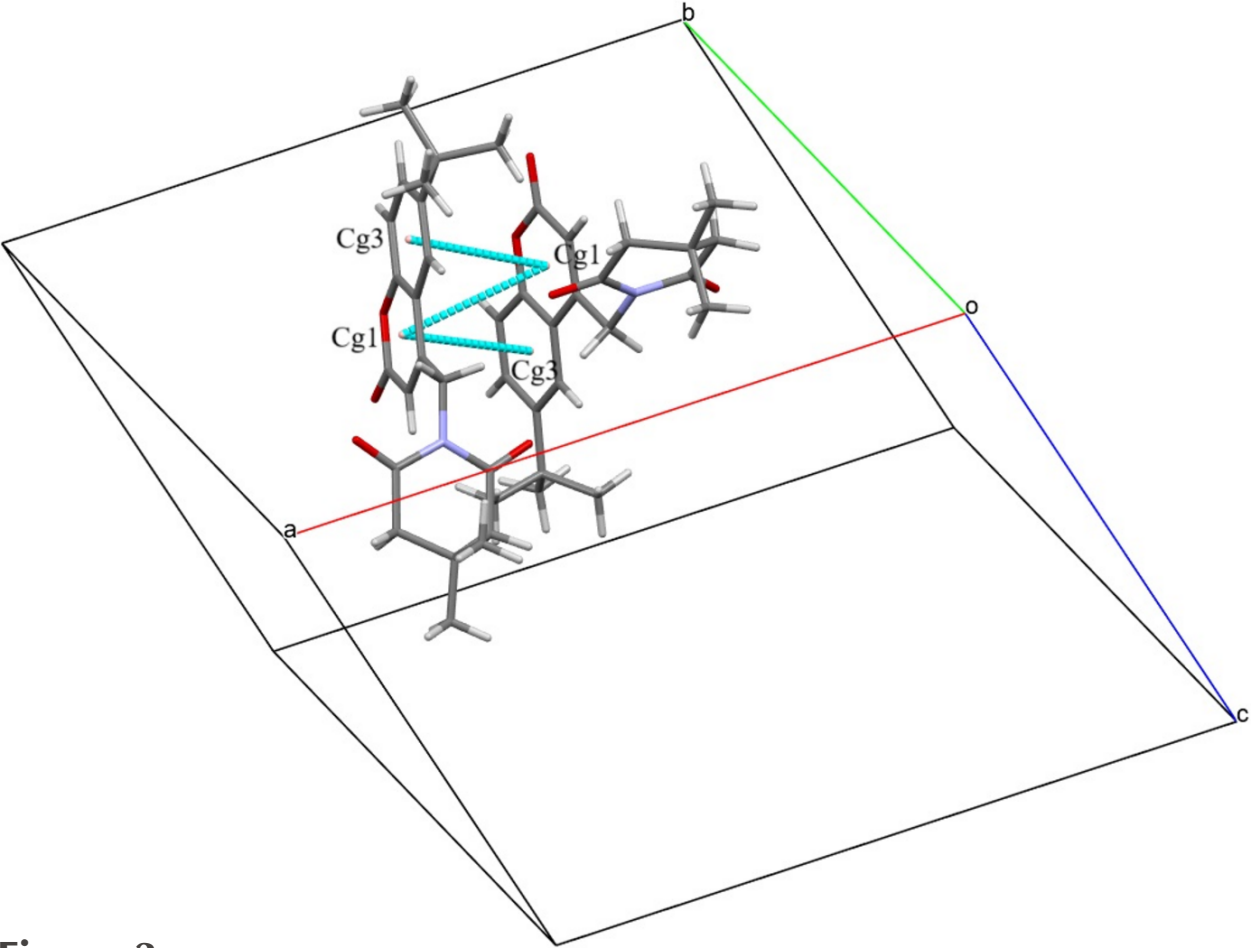
The mol­ecular packing of (I) showing the π–π stacking.

In the crystal, C2—H2⋯O2 and C14—H14*A*⋯O3 interactions (Fig. 2 ▸*a*,*b*, Table 1 ▸) leads to the formation of head-to-head dimers with an 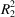
(8) graph-set motif and 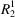
(9) and 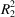
(10) ring motifs (Bernstein *et al.*, 1995 ▸) along [001] and [100] , respectively. The C16—H16*A*⋯O2 inter­action connects the mol­ecules in the [010] direction. The mol­ecular packing is further consolidated by π–π stacking [centroid–centroid distances *Cg*1⋯*Cg*1 = 3.885 (2) and *Cg*1⋯*Cg*3 = 3.738 (2) Å, where *Cg*1 and *Cg*3 are the centroids of the C1–C3/O1/C4/C9 and C4–C9 rings, respectively] as shown in Fig. 3 ▸.

## 4. Hirshfeld surface analysis

**Figure 4:**
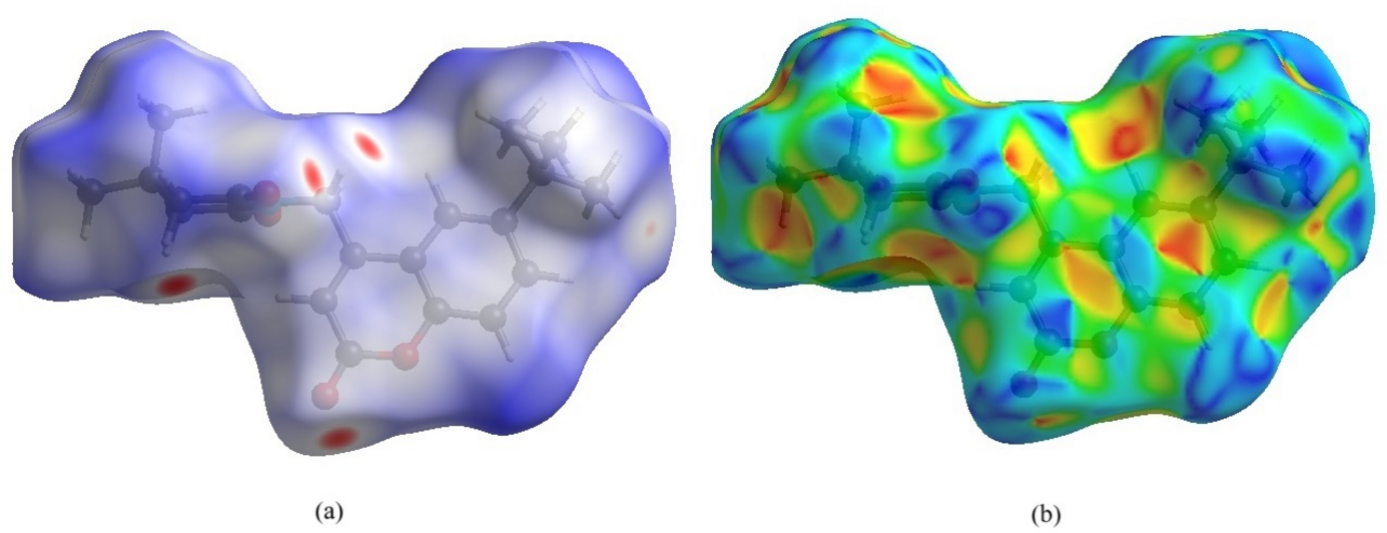
The Hirshfeld surface of the title mol­ecule mapped over (*a*) *d*_norm_ and (*b*) shape-index.

**Figure 5:**
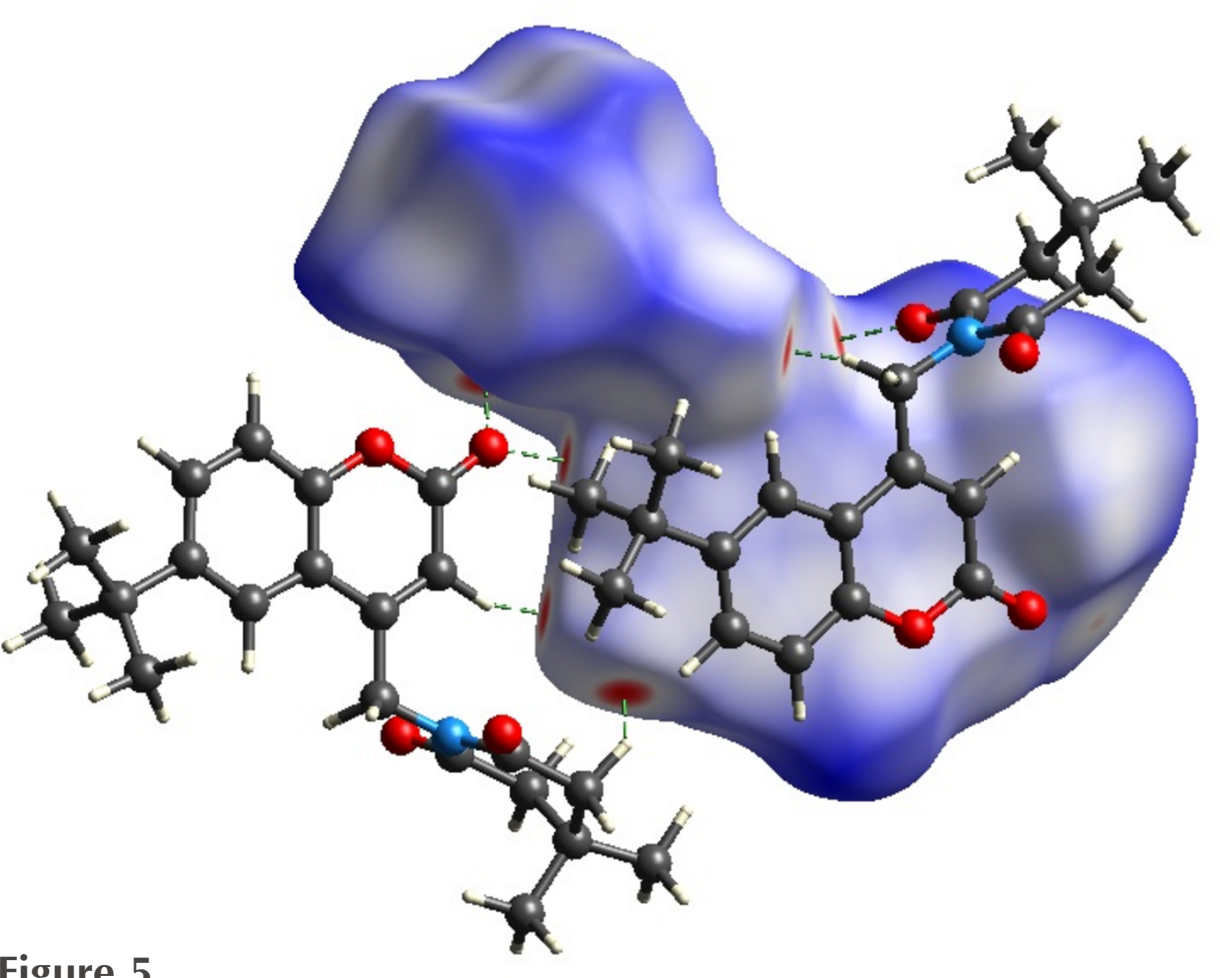
The Hirshfeld surface mapped over *d*_norm_ showing the C—H⋯O inter­actions generating 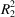
(8), and 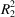
(10) synthons.

**Figure 6:**
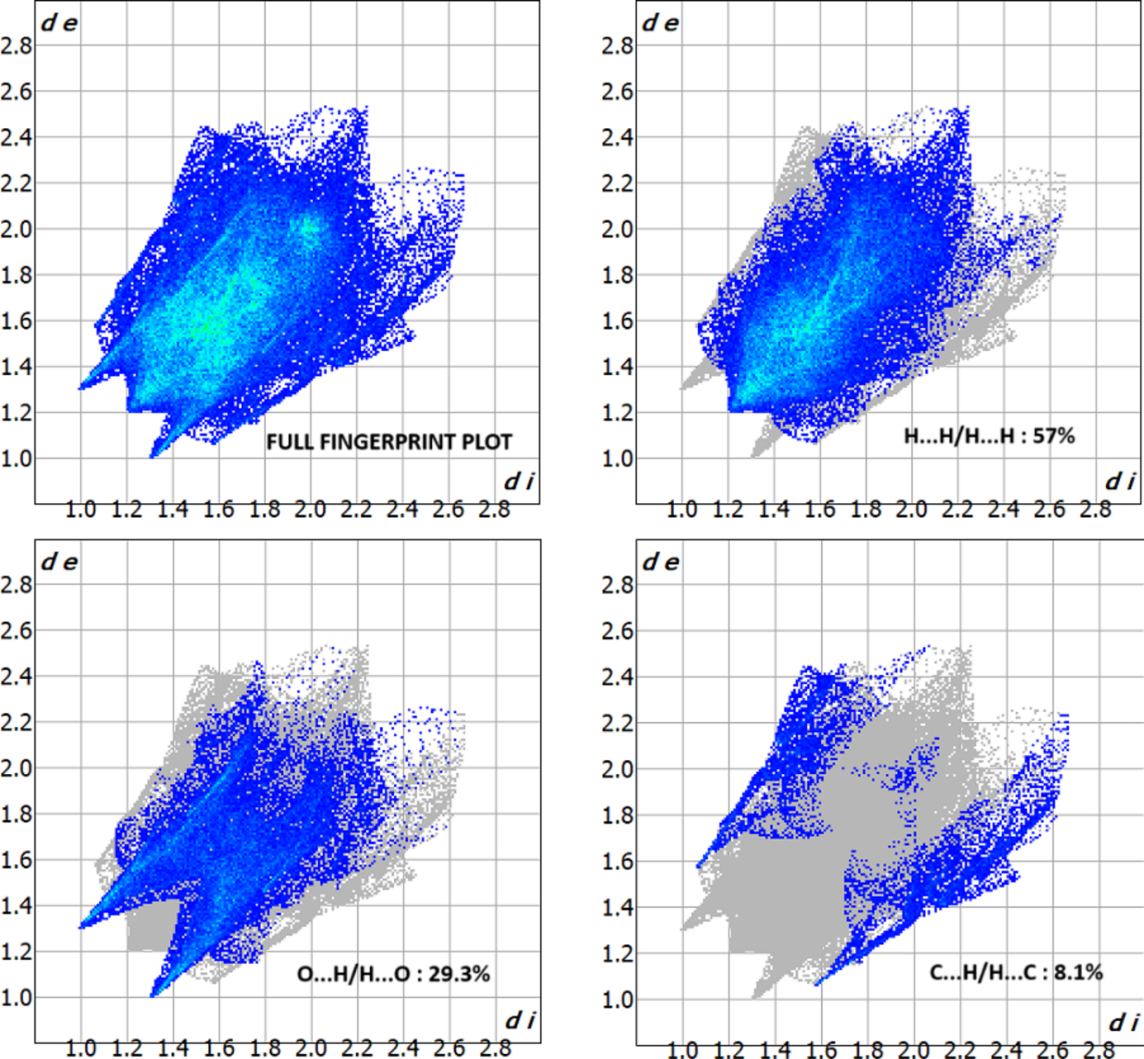
Two-dimensional fingerprint plots for the title compound, showing all inter­actions, and delineated into H⋯H, C⋯H/H⋯C and H⋯O/O⋯H inter­actions.

**Figure 7:**
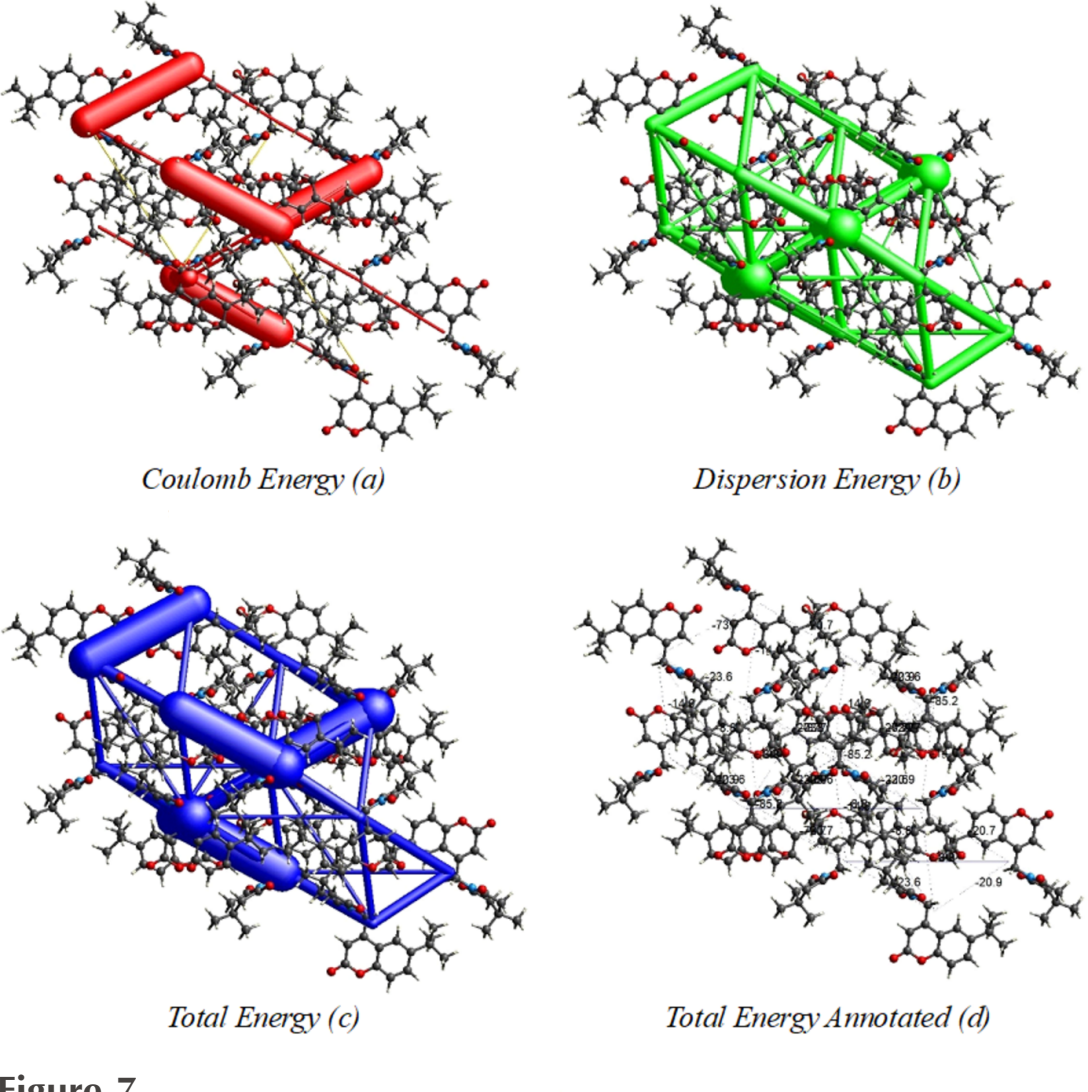
Energy frameworks calculated for the title compound, showing (*a*) Coulomb force, (*b*) dispersion force and (*c*), (*d*) total energy diagrams. The cylindrical radii are proportional to the relative strength of the corresponding energies; they were adjusted to a cutoff value of 5 kJ mol^−1^.

*CrystalExplorer17.5* (Turner *et al.*, 2017 ▸) was used to perform a Hirshfeld surface (Hirshfeld, 1977 ▸; Spackman & Jayatilaka, 2009 ▸) analysis to qu­antify the various inter­molecular inter­actions of the title mol­ecule. The Hirshfeld surface mapped over the normalized contact distance *d_norm_* is shown in Fig. 4 ▸*a*. Contacts with distances equal to the sum of the van der Waals radii are indicated in white, while those with shorter or longer distances are represented in red and blue, respectively. The shape-index detects even minor variations in surface shape. It shows the electron-density surface surrounding mol­ecular inter­actions. The very small range of light colours on the surface signifies a weaker and longer inter­action other than hydrogen bonds. The presence of red and blue triangles on the surface of the shape index is evidence of π–π inter­actions, as shown in Fig. 4 ▸*b*. Fig. 5 ▸ shows the Hirshfeld surface where hydrogen-bonding inter­actions with neighbouring mol­ecules occur at the red spots. The fingerprint plots in Fig. 6 ▸ indicate that the major contributions to the crystal structure are from H⋯H (57.0%), O⋯H/H⋯O (29.3%) and C⋯H/H⋯C (8.1%) contacts. The characteristic spikes in the O⋯H/H⋯O plot indicate the presence of hydrogen bonds listed in Table 1 ▸. The net inter­action energies are *E*_ele_ =−267.7 kJ mol^−1^, *E*_pol_ = −43.6 kJmol^−1^, *E*_dis_ = −267.7 kJ mol^−1^, *E*_rep_ = 170.2 kJ mol^−1^ and total inter­action energy *E*_tot_ = 128.8 kJ mol^−1^. The topology of the energy frameworks related to (*a*) Coulombic energy, (*b*) dispersion energy and (*c*) total energy inter­actions viewed along *a*-axis is shown in Fig. 7 ▸, where the total energy annotated (*d*) is also shown.

## 5. Density functional studies

**Figure 8:**
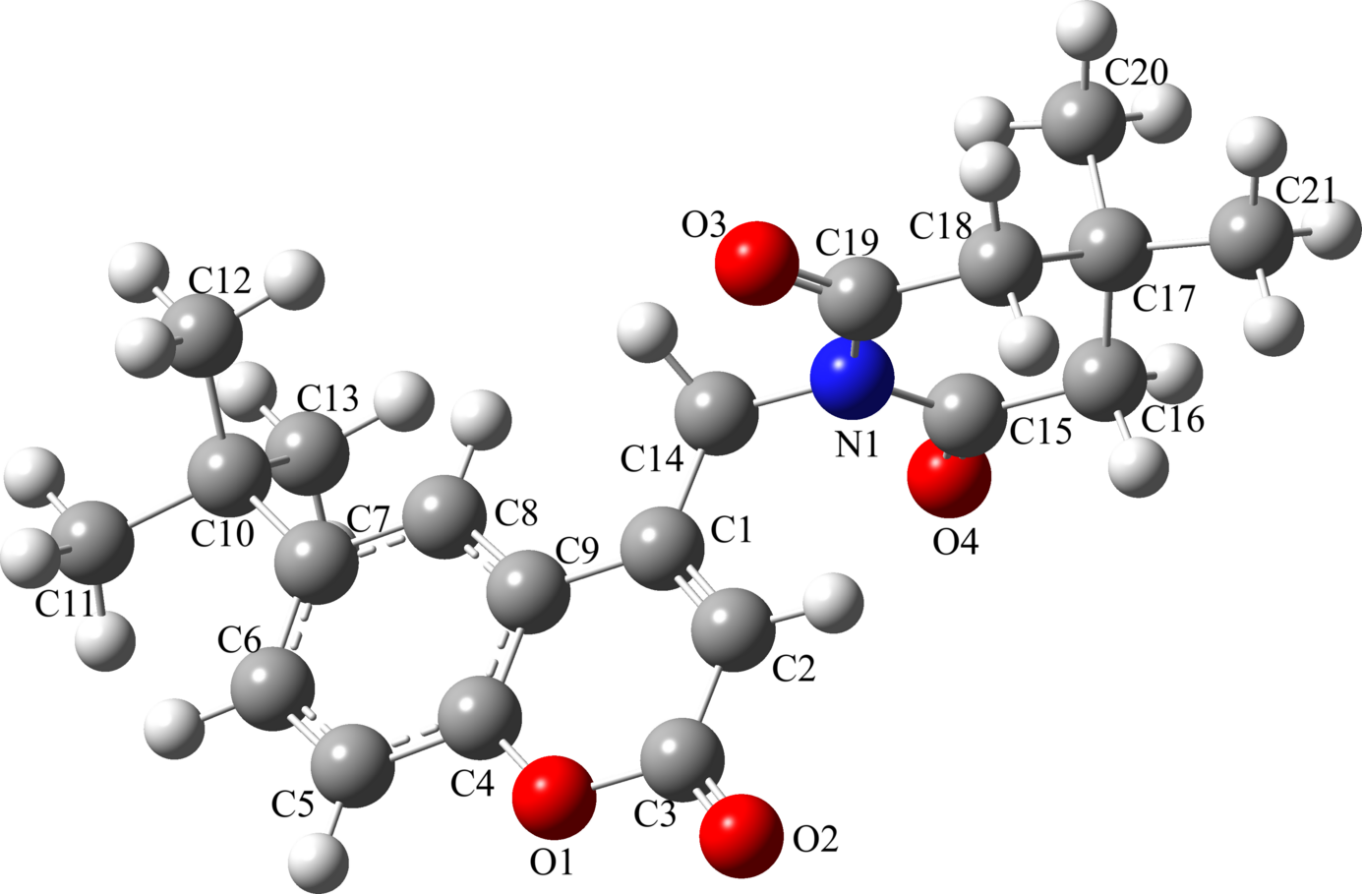
The DFT=optimized structure of the title compound.

**Figure 9:**
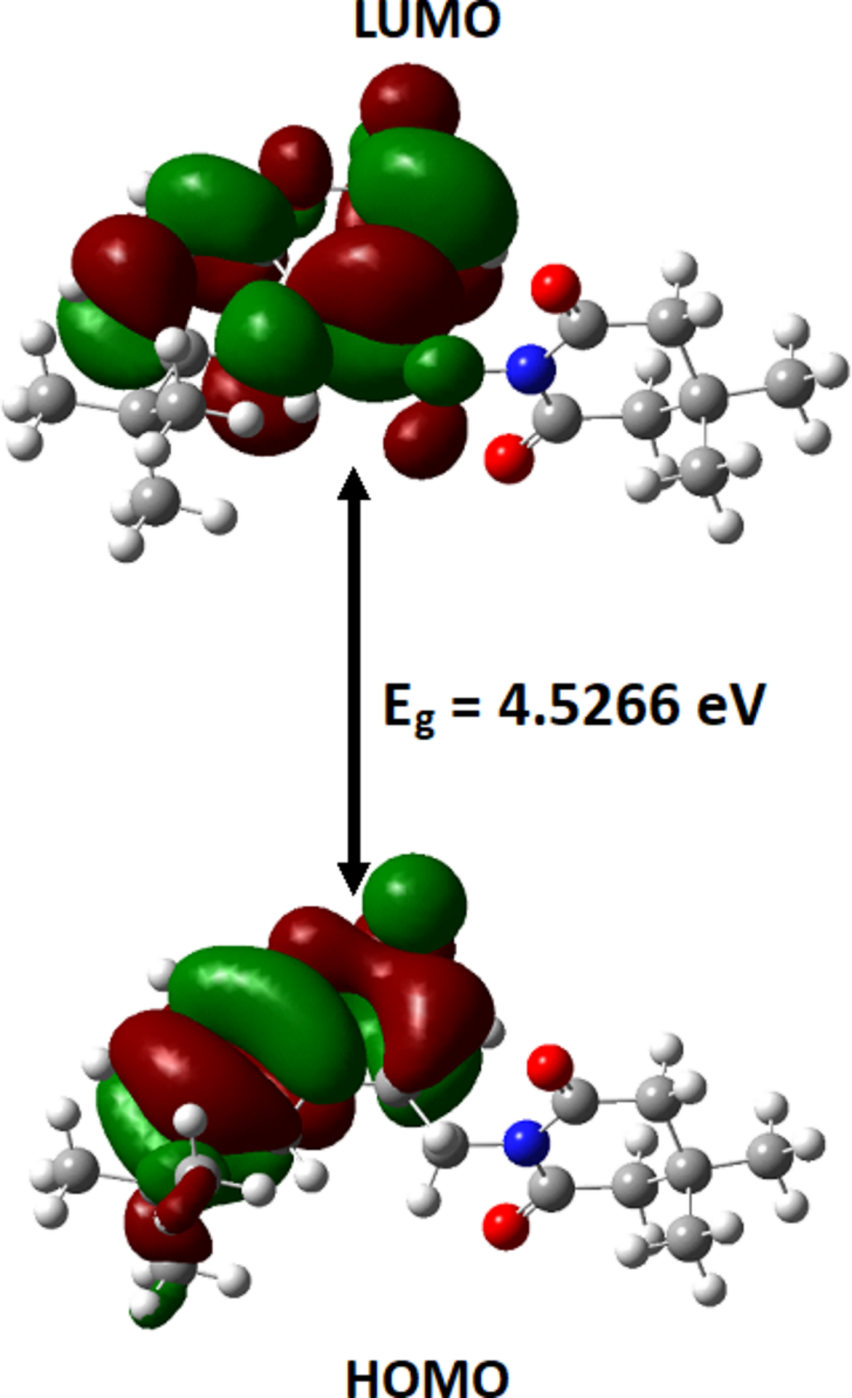
HOMO and LUMO of compound (I) with the energy band gap.

DFT studies were performed in the gas phase at the B3LYP/6-311+ G(d,p) level using *Gaussian 09W* (Frisch *et al.*, 2009 ▸). *GaussView 5.0* was used to generate the optimized structure of the mol­ecule shown in Fig. 8 ▸. The optimized bond parameters obtained are in good agreement with those obtained from SCXRD analysis (Table 2 ▸). The small deviations observed may be attributed to the fact that theoretical calculations were performed in the gas phase whereas the SCXRD measurements are made in the solid state. The frontier mol­ecular orbitals HOMO and LUMO generated using DFT calculations are −6.59 eV and −2.06 eV, respectively. The energy gap is 4.5366 eV (Fig. 9 ▸). The reactivity descriptors calculated from the energy gap value, *viz*. ionization energy (*I*), electron affinity (*A*), electronegativity (χ), chemical hardness (η), chemical potential (μ), electrophilicity index (ω) and chemical softness (*S*) are 6.59, 2.06, 4.325, 2.65, −4.325, 4.129 eV and 0.221 eV^−1^, respectively. The electrophilicity index value indicates the mol­ecule exhibits strong electrophilicity.

**Figure 10:**
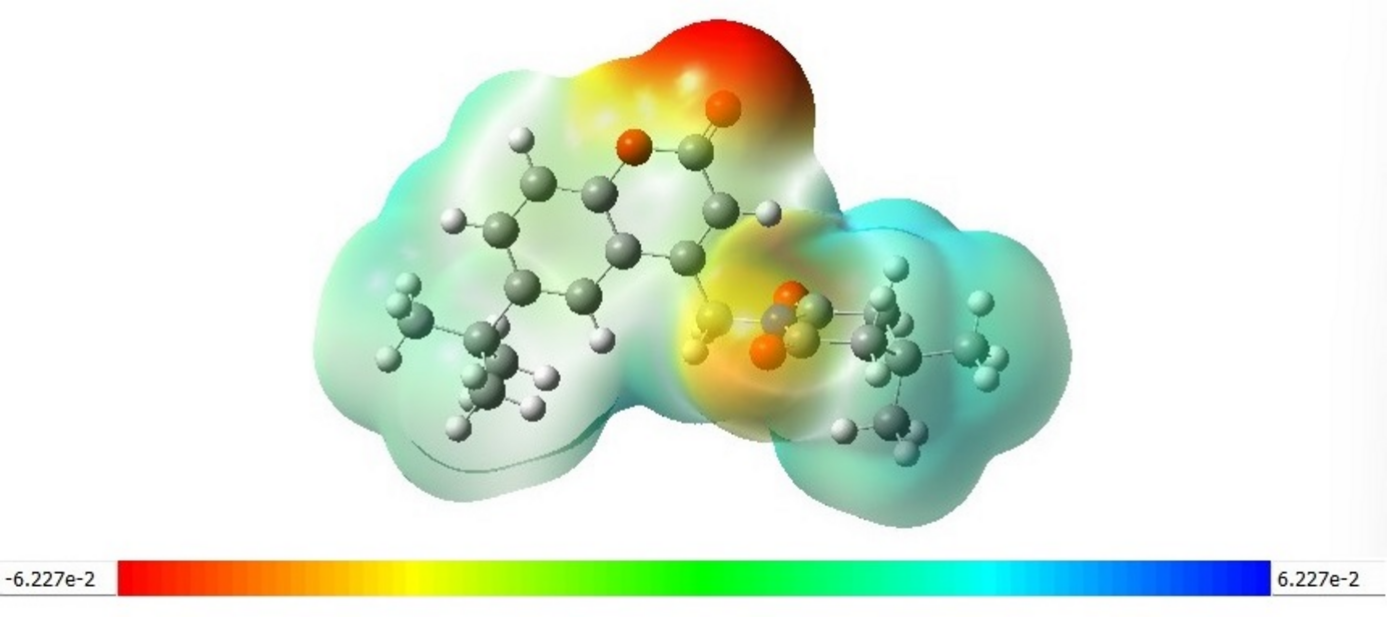
MEP plots of the title compound; regions of attractive potential appear in red and those of repulsive potential appear in blue.

The MEP surface of the optimized structure of the title compound is depicted in Fig. 10 ▸. Nucleophilic reactive sites of the mol­ecule are represented by red regions on the MEP surface. In the MEP surface for the title compound, the red around the oxygen atom of the coumarin fragment shows it is an active site for nucleophilic inter­actions.

## 6. Mol­ecular docking

**Figure 11:**
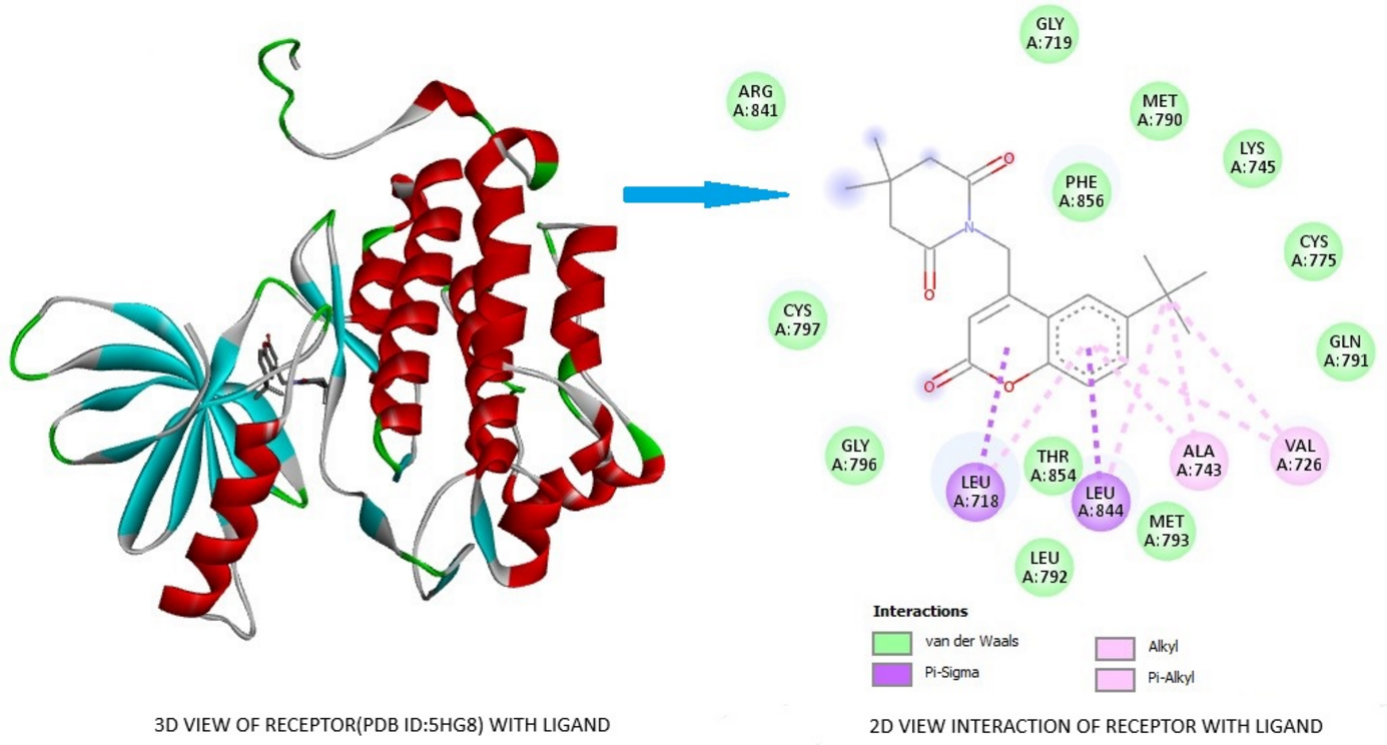
A three-dimensional view of the lung cancer epidermal growth factor receptor (EGFR) (PDBID: 5HG8) protein and two-dimensional view of the mol­ecular inter­actions between the ligand and amino acid residues.

The lung cancer epidermal growth factor receptor (EGFR; PDBID: 5HG8) and breast cancer carbonic anhydrase IX (CAIX; PDBID: 6NLV) proteins were selected as receptors with the title compound as a ligand. *AutoDock Vina* (Morris *et al.*, 2009 ▸) was used to carry out the docking studies in both cases. Good binding affinity scores of −9.5 and −8.2 kcal mol^−1^, respectively, were obtained for the lung and breast cancer receptors respectively. The inter­action as generated by *Discovery Studio Visualizer* (Biovia, 2017 ▸) for EGFR and the title ligand is shown in Fig. 11 ▸. It clearly illustrates that there are two π–σ inter­actions between the centroid *Cg*1 with the amino acid LEU A:718 and *Cg*3 with the amino acid LEU A:844. *Cg*3 acts as an anchor point for the amino acids LEU A:718, LEU A:844, ALA A:743 and VAL A:726, forming π–alkyl inter­actions. In addition there are three alkyl bonds and twelve van der Waals inter­actions between the ligand and the amino acid residues of the protein.

**Figure 12:**
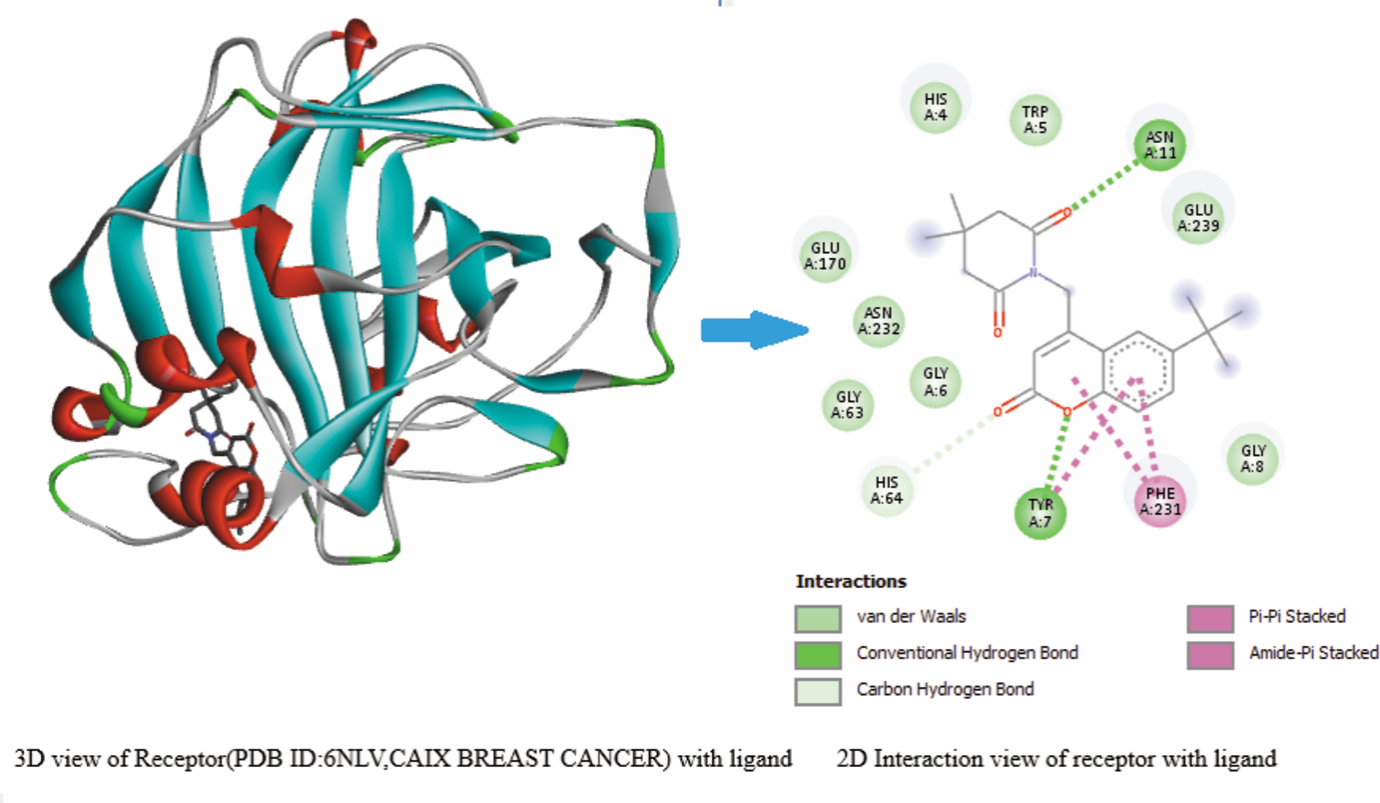
A three-dimensional view of the breast cancer carbonic anhydrase IX (CAIX)(PDBID:6 NLV) protein and two-dimensional view of the mol­ecular inter­actions between the ligand and amino acid residues.

The inter­actions generated between the breast cancer carbonic anhydrase IX protein and the title ligand is shown in Fig. 12 ▸. There are two conventional hydrogen bonds with amino acids ASN A:11 and TYR A:7 and the oxygen atoms of the piperidine and coumarin fragments. *Cg*1 and *Cg*3 act as anchor points for the PHE A:231 and TYR A:7 amino acids, forming π–π stacking inter­actions. Hydrogen bonding is observed with with amino acid HIS A:64 and the ligand is also enclosed by nine van der Waals inter­actions. Hence, the title mol­ecule can be considered as a potential candidate for lung cancer and breast cancer applications. The efficiency of the ligand was tested practically by carrying out biological studies as detailed below.

## 7. Biological studies

**Figure 13:**
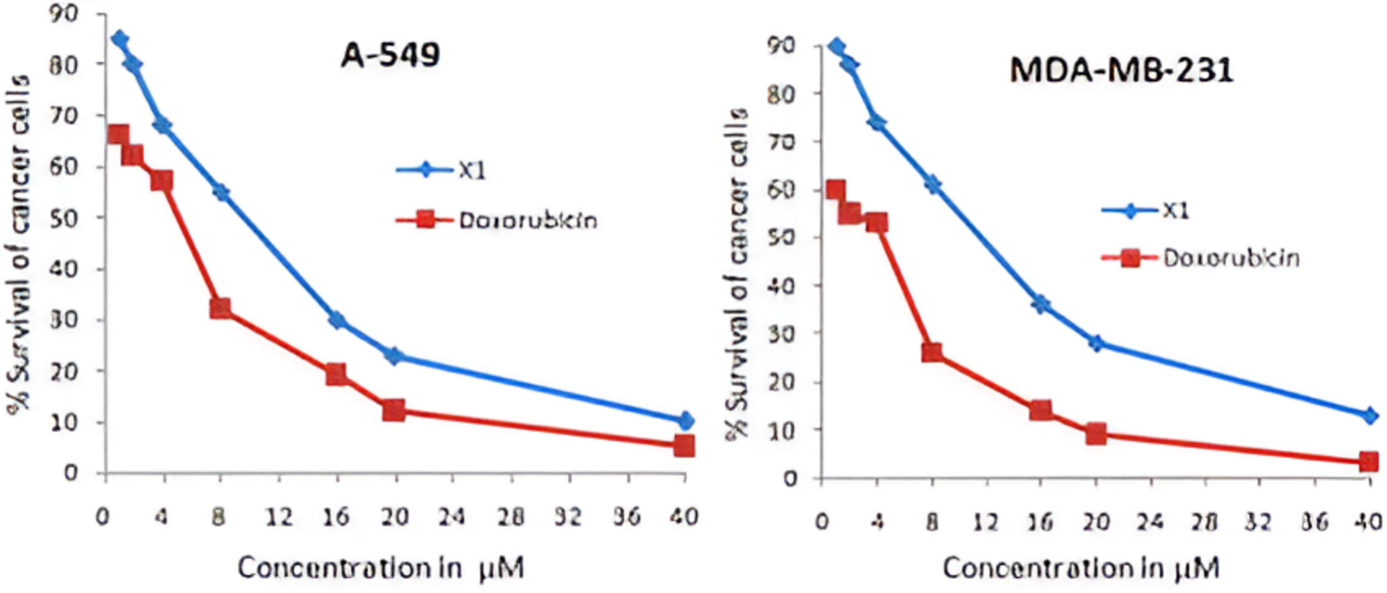
The concentration-effect curves of active compound (I) in A-549 and MDA-MB-231 cell lines.

The anti-cancer activity of the title compound was evaluated against two human cancer cell lines, A-549 (human lung carcinoma) and MDAMB-231 (human adenocarcinoma mammary gland), by MTT assay (Zheng *et al.*, 2012 ▸; Takla *et al.*, 2023 ▸). The title compound inhibited cell proliferation with IC_50_ values of 9.34 µ*M* and 11.57 µ*M*, respectively, as compared values for the standard drug doxorubicin IC_50_ = 5.13 and 4.82 µ*M*, respectively. The concentration-effect curves of for the title compound against A-549 and MDA-MB-231 cell lines are shown in Fig. 13 ▸**.**

Furthermore, in order to check the safety profile, the title compound was tested for cytotoxicity on HEK293 cell lines (Yadagiri *et al.*, 2014 ▸). In the case of the A549 and MDA-MB-231cancer cell lines, it showed a good safety profile on HEK293 with selectivity indices (SI) of 7.97 and 6.45, respectively. The results for anti­cancer activity against cell lines A-549 and MDA-MB-231are shown in Table 3 ▸. Overall it was found that the compound exhibited low toxicity against the HEK293 cell line with an IC_50_ value of 71.03 µ*M*. These data will help in further optimization of conjugates of the title compound to obtain more potent and safer anti-cancer agents with enhanced properties.

## 8. Database survey

A search in the Cambridge Crystallographic Database (CSD version 2.0.4 of December 2019; Groom *et al.*. 2016 ▸) for mol­ecules containing the butyl-2-oxo-chromene fragment resulted in one match. EFUVUY (He *et al.*, 2014 ▸) is very similar compared to the title compound with a dihedral angle of 0.17° between the aromatic rings of the ten-membered oxo-chromene fragment. A search for mol­ecules containing the butyl-2*H*-chromene moiety resulted in another hit, *viz*. FABCEU (Duong *et al.*, 2020 ▸), which is similar to the title compound in having a dihedral angle of 1.24° between the aromatic rings of the ten-membered oxo-chromene fragments. In general, the ten-membered ring system is nearly planar. A search for mol­ecules containing the oxo-2*H*-chromene moiety gave more than thirty hits. Among these, AFOQET (Abou *et al.*, 2013 ▸), AGAREH (Bibila Mayaya Bisseyou *et al.*, 2013 ▸) and AYOXAO (Abou *et al.*, 2011 ▸) have simple substitutions at the *ortho* position of the aromatic ring of the oxo-2*H*-chromene, the torsion angles at the linked substitution being 175.56, 180.0 and −175.3°, respectively. In the title compound, the comparable angle is −93.71 (16)°.

## 9. Synthesis and crystallization

The title mol­ecule was synthesized using an S_N_2 reaction of bromo­methyl coumarin with 4,4-di­methyl­piperidine-2,6-dione.

### 9.1. Synthesis of ethyl 4-bromo­acetyl acetate

Ethyl aceto­acetate (**i**) (0.38mol) was mixed with dry ether (60 ml), stirred for 10 minutes, after that, the reaction mixture was cooled to 273–278 K. Maintaining that temperature, liquid bromine (20.5 ml, 0.38mol) was slowly added to the reaction mixture, and stirring continued at room temperature for 24 h. The reaction mixture was then decomposed into crushed ice, the ether layer was separated, washed with distilled water and dried over anhydrous calcium chloride to obtain the product ethyl 4-bromo­aceto­acetate (**ii**).

### 9.2. Synthesis of 4-bromo­methyl-6-*tert*-butyl-2*H*-chromen-2-one

Ethyl 4-bromo­aceto­acetate (0.1 *M*) and 4-*tert*-butyl­phenol (0.1 *M*) were taken in a round-bottom flask and cooled to 273–278 K. Concentrated sulfuric acid (35 ml) was added slowly, maintaining the temperature at 273–278 K. The solution was then stirred for 24 h at room temperature. A deep-red solution was formed at the end of the reaction, and then it was poured into the crushed ice. The precipitate of 4-bromo­methyl-6-*tert*-butyl-2*H*-chromen-2-one (**iii**) was filtered and washed with water and ethanol.

### 9.3. Synthetic procedure to prepare the title compound (I)

4-Bromo­methyl-6-*tert*-butyl-2*H*-chromen-2-one (**iii)** (0.001 mol) and 4,4-di­methyl­piperidine-2,6-dione (**iv**) (0.001 mol) and 5 ml of dry acetone were taken in a round-bottom flask. Then 0.003 mol of K_2_CO_3_ were added and the reaction mixture was refluxed at 328–338 K for 10 h. Formation of the compound was monitored by TLC. After completion of the reaction, it was poured onto crushed ice, and the product was washed with water to remove excess K_2_CO_3_ and dried to obtain the title compound at room temperature. Fine crystals were obtained by the slow evaporation technique using DMF as a solvent.

1-[(6-*tert*-butyl-2-oxo-2*H*-chromen-4-yl)meth­yl]-4,4-di­meth­yl­piperidine-2,6-dione: Off-white solid; m.p. 546–547 K; Yield: 2.71 g (82.37%). ^1^H NMR (400 MHz, CDCl_3_, δ ppm): 1.15 (*s*, 6H, –CH_3_), 1.34 (*s*, 9H, –CH_3_), 2.64 (*s*, 4H, –CH_2_), 5.16 (*s*, 2H, –CH_2_), 5.95 (*s*, 1H, –CH), 7.24–7.28 (*m*, 2H, Ar-H), 7.57–7.60 (*m*, 1H, Ar-H); ^13^C NMR (100 MHz, CDCl_3_, δ ppm): 28.07, 29.46, 31.47, 34.83, 39.33, 46.27, 111.95, 116.97, 117.21, 119.82, 129.88, 147.53, 149.95, 151.65, 160.91, 171.70; GC-MS: 355 [*M*]^+^. Micro elemental analysis calculated for C_21_H_25_NO_4_ (*M*_r_ 355.43) C, 70.96; H, 7.09; N, 3.94; O, 18.01%, found C, 70.99; H, 7.11; N, 3.97%.
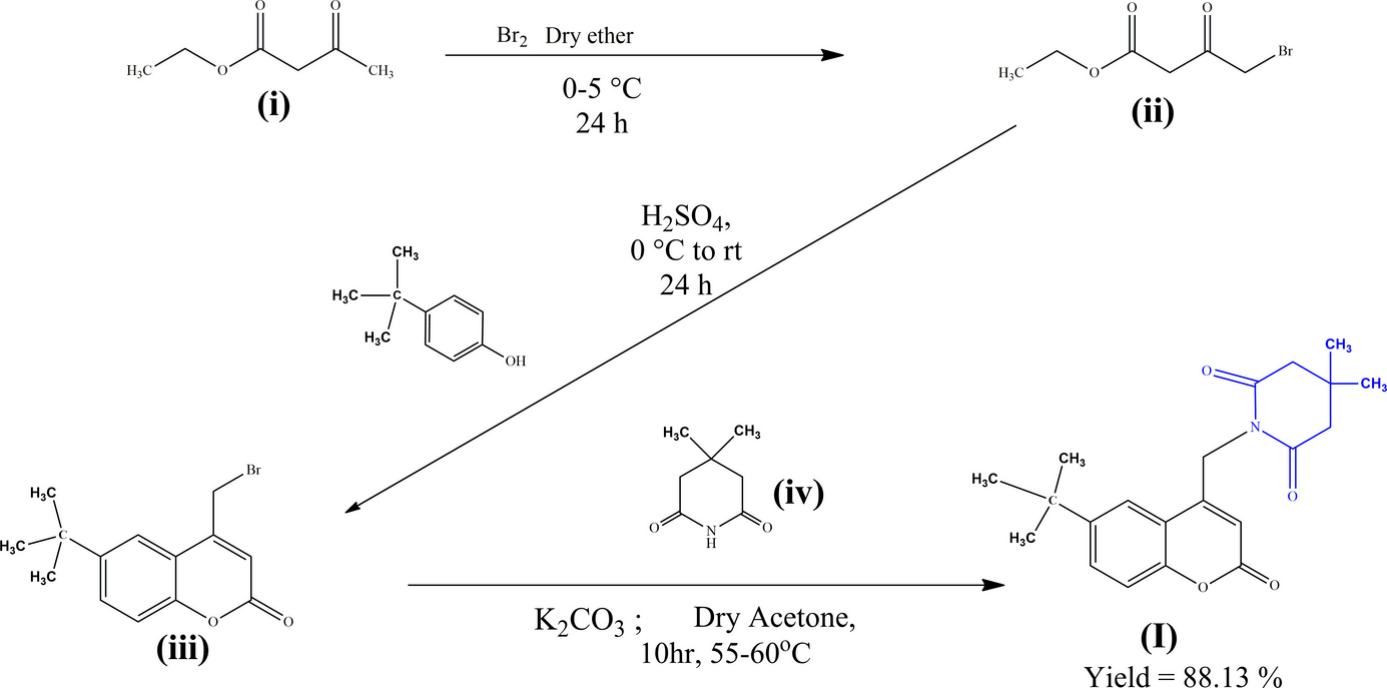


## 10. Refinement

Crystal data, data collection and structure refinement details are summarized in Table 4 ▸. H atoms were positioned with idealized geometry and refined using a riding model with C—H = 0.93–0.97 Å and *U*_iso_(H) = 1.2–1.5*U*_eq_(C).

## Supplementary Material

Crystal structure: contains datablock(s) I. DOI: 10.1107/S2056989025001550/nx2021sup1.cif

Structure factors: contains datablock(s) I. DOI: 10.1107/S2056989025001550/nx2021Isup2.hkl

Supporting information file. DOI: 10.1107/S2056989025001550/nx2021Isup3.cml

CCDC reference: 2425502

Additional supporting information:  crystallographic information; 3D view; checkCIF report

## Figures and Tables

**Table 1:** Hydrogen-bond geometry (Å, °)

*D*—H⋯*A*	*D*—H	H⋯*A*	*D*⋯*A*	*D*—H⋯*A*
C16—H16*A*⋯O2^i^	0.97	2.41	3.1800 (19)	136
C14—H14*A*⋯O3^ii^	0.97	2.49	3.3898 (18)	155
C2—H2⋯O2^i^	0.93	2.50	3.3687 (18)	155
C14—H14*A*⋯O3	0.97	2.40	2.6915 (19)	97
C14—H14*B*⋯O4	0.97	2.34	2.698 (2)	101

Symmetry codes: (i) 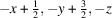; (ii) 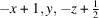.

**Table 2:** Selected bond lengths, angles and torsion angles (Å, °)

Parameter	SCXRD	DFT
O1—C3	1.3732 (18)	1.3885
O1—C4	1.3798 (17)	1.3885
O3—C19	1.2176 (18)	1.2121
O4—C15	1.212 (2)	1.212
N1—C14	1.4627 (18)	1.466
C3—O1—C4	121.19 (12)	122.13
C19—N1—C15	124.25 (12)	120.5
O1—C3—C2	117.62 (13)	116.10
C21—C17—C20	109.87 (13)	109.41
C15—N1—C14—C1	−93.71 (16)	−92.45
C19—N1—C15—O4	−178.11 (15)	−177.35

**Table 3:** Experimental details of cytotoxicity (IC_50_) against cell lines A-549 and MDA-MB-231 (μ*M*)

Product/Cell lines	Title compound	Doxorubicin
A-549	9.34±0.68	5.13±0.41
MDA-MB-231	11.57±0.54	4.82±0.38
HEK293	71.03	86.47
SI for A-549	7.97	16.85
SI for MDA-MB-231	6.45	18.71

**Table 4:** Experimental details

Crystal data	
Chemical formula	C_21_H_25_NO_4_
*M*_r_	355.42
Crystal system, space group	Monoclinic, *C*2/*c*
Temperature (K)	296
*a*, *b*, *c* (Å)	17.9534 (7), 15.8109 (7), 14.6429 (6)
β (°)	113.189 (2)
*V* (Å^3^)	3820.7 (3)
*Z*	8
Radiation type	Mo *K*α
μ (mm^−1^)	0.09
Crystal size (mm)	0.23 × 0.21 × 0.17

Data collection	
Diffractometer	Bruker *SMART* APEXII CCD
Absorption correction	Multi-scan (*SADABS*; Krause et al., 2015 ▸)
*T*_min_, *T*_max_	0.980, 0.985
No. of measured, independent and observed [*I* > 2σ(*I*)] reflections	22459, 3365, 2922
*R*_int_	0.042
(sin θ/λ)_max_ (Å^−1^)	0.595

Refinement	
*R*[*F*^2^ > 2σ(*F*^2^)], *wR*(*F*^2^), *S*	0.043, 0.111, 1.06
No. of reflections	3365
No. of parameters	240
H-atom treatment	H-atom parameters constrained
Δρ_max_, Δρ_min_ (e Å^−3^)	0.26, −0.32

Computer programs: *APEX2* and *SAINT* (Bruker, 2014 ▸), *SHELXT* (Sheldrick, 2015*a* ▸), *SHELXL* (Sheldrick, 2015*b* ▸), *Mercury* (Macrae *et al.*, 2020 ▸) and *SHELXTL* (Sheldrick, 2008 ▸).

## supplementary crystallographic information

### 1-[(6-tert-Butyl-2-oxo-2H-chromen-4-yl)methyl]-4,4-dimethylpiperidine-2,6-dione Crystal data

**Table:**

C_21_H_25_NO_4_	*F*(000) = 1520
*M**_r_* = 355.42	*D*_x_ = 1.236 Mg m^−^^3^
Monoclinic, *C*2/*c*	Melting point: 546 K
Hall symbol: -C 2yc	Mo *K*α radiation, λ = 0.71073 Å
*a* = 17.9534 (7) Å	Cell parameters from 2922 reflections
*b* = 15.8109 (7) Å	θ = 2.6–26.0°
*c* = 14.6429 (6) Å	µ = 0.09 mm^−^^1^
β = 113.189 (2)°	*T* = 296 K
*V* = 3820.7 (3) Å^3^	BLOCK, colourless
*Z* = 8	0.23 × 0.21 × 0.17 mm

### 1-[(6-tert-Butyl-2-oxo-2H-chromen-4-yl)methyl]-4,4-dimethylpiperidine-2,6-dione Data collection

**Table:**

Bruker SMART APEXII CCD diffractometer	3365 independent reflections
Radiation source: fine-focus sealed tube	2922 reflections with *I* > 2σ(*I*)
Graphite monochromator	*R*_int_ = 0.042
Detector resolution: 0.97 pixels mm^-1^	θ_max_ = 25.0°, θ_min_ = 2.6°
φ and Ω scans	*h* = −21→21
Absorption correction: multi-scan (SADABS; Krause et al., 2015)	*k* = −18→18
*T*_min_ = 0.980, *T*_max_ = 0.985	*l* = −17→17
22459 measured reflections	

### 1-[(6-tert-Butyl-2-oxo-2H-chromen-4-yl)methyl]-4,4-dimethylpiperidine-2,6-dione Refinement

**Table:**

Refinement on *F*^2^	Primary atom site location: structure-invariant direct methods
Least-squares matrix: full	Secondary atom site location: difference Fourier map
*R*[*F*^2^ > 2σ(*F*^2^)] = 0.043	Hydrogen site location: inferred from neighbouring sites
*wR*(*F*^2^) = 0.111	H-atom parameters constrained
*S* = 1.06	*w* = 1/[σ^2^(*F*_o_^2^) + (0.0469*P*)^2^ + 4.1402*P*] where *P* = (*F*_o_^2^ + 2*F*_c_^2^)/3
3365 reflections	(Δ/σ)_max_ < 0.001
240 parameters	Δρ_max_ = 0.26 e Å^−^^3^
0 restraints	Δρ_min_ = −0.32 e Å^−^^3^
0 constraints	

### 1-[(6-tert-Butyl-2-oxo-2H-chromen-4-yl)methyl]-4,4-dimethylpiperidine-2,6-dione Special details

**Table:**

Geometry. All esds (except the esd in the dihedral angle between two l.s. planes) are estimated using the full covariance matrix. The cell esds are taken into account individually in the estimation of esds in distances, angles and torsion angles; correlations between esds in cell parameters are only used when they are defined by crystal symmetry. An approximate (isotropic) treatment of cell esds is used for estimating esds involving l.s. planes.

### 1-[(6-tert-Butyl-2-oxo-2H-chromen-4-yl)methyl]-4,4-dimethylpiperidine-2,6-dione Fractional atomic coordinates and isotropic or equivalent isotropic displacement parameters (Å^2^)

**Table:**

	*x*	*y*	*z*	*U*_iso_*/*U*_eq_	
O1	0.38958 (6)	0.85989 (6)	0.21647 (8)	0.0234 (3)	
O2	0.30944 (7)	0.84959 (7)	0.05798 (8)	0.0290 (3)	
O3	0.42605 (6)	0.53779 (7)	0.10830 (8)	0.0286 (3)	
O4	0.22270 (8)	0.55466 (9)	0.21639 (10)	0.0441 (4)	
N1	0.32502 (7)	0.54449 (8)	0.16368 (9)	0.0203 (3)	
C10	0.56284 (9)	0.71989 (10)	0.60161 (11)	0.0240 (3)	
C3	0.34437 (9)	0.81208 (10)	0.13520 (11)	0.0219 (3)	
C2	0.34194 (9)	0.72165 (9)	0.14891 (11)	0.0212 (3)	
H2	0.312072	0.688242	0.094492	0.025*	
C1	0.38126 (8)	0.68406 (9)	0.23737 (11)	0.0191 (3)	
C8	0.47121 (8)	0.70433 (10)	0.41872 (10)	0.0202 (3)	
H8	0.470639	0.646417	0.429532	0.024*	
C7	0.51444 (9)	0.75663 (10)	0.49805 (11)	0.0211 (3)	
C6	0.51574 (9)	0.84311 (10)	0.47904 (11)	0.0253 (4)	
H6	0.545096	0.879191	0.530918	0.030*	
C5	0.47458 (9)	0.87654 (10)	0.38523 (12)	0.0250 (3)	
H5	0.476491	0.934270	0.374167	0.030*	
C4	0.43057 (8)	0.82311 (9)	0.30815 (11)	0.0194 (3)	
C9	0.42829 (8)	0.73610 (9)	0.32264 (10)	0.0183 (3)	
C11	0.57278 (15)	0.78452 (14)	0.68234 (14)	0.0559 (6)	
H11A	0.605448	0.830688	0.676721	0.084*	
H11B	0.598680	0.758392	0.746323	0.084*	
H11C	0.520489	0.805330	0.674996	0.084*	
C13	0.52054 (17)	0.64368 (17)	0.62166 (15)	0.0770 (10)	
H13A	0.465509	0.658110	0.609532	0.115*	
H13B	0.548167	0.626427	0.689603	0.115*	
H13C	0.521116	0.598151	0.578562	0.115*	
C12	0.64529 (13)	0.69548 (19)	0.60640 (15)	0.0654 (8)	
H12A	0.639723	0.652431	0.557906	0.098*	
H12B	0.677532	0.674276	0.671500	0.098*	
H12C	0.671242	0.744163	0.592851	0.098*	
C14	0.37878 (9)	0.58983 (9)	0.25218 (11)	0.0233 (3)	
H14A	0.433176	0.567330	0.272170	0.028*	
H14B	0.361162	0.579367	0.305851	0.028*	
C19	0.35637 (9)	0.52013 (9)	0.09426 (11)	0.0205 (3)	
C18	0.30041 (9)	0.47477 (10)	0.00280 (11)	0.0227 (3)	
H18A	0.332310	0.436947	−0.019585	0.027*	
H18B	0.275426	0.516128	−0.049278	0.027*	
C17	0.23360 (9)	0.42360 (9)	0.01691 (11)	0.0236 (3)	
C16	0.19056 (9)	0.48305 (10)	0.06247 (12)	0.0256 (4)	
H16A	0.160169	0.524315	0.012729	0.031*	
H16B	0.151960	0.450402	0.079227	0.031*	
C15	0.24523 (10)	0.52923 (10)	0.15344 (12)	0.0256 (4)	
C21	0.17368 (11)	0.39218 (11)	−0.08396 (12)	0.0349 (4)	
H21A	0.150430	0.439691	−0.126479	0.052*	
H21B	0.131474	0.360311	−0.075142	0.052*	
H21C	0.201473	0.356785	−0.113621	0.052*	
C20	0.26966 (11)	0.34836 (10)	0.08600 (13)	0.0341 (4)	
H20A	0.297280	0.311900	0.057153	0.051*	
H20B	0.227007	0.317498	0.095009	0.051*	
H20C	0.307318	0.368498	0.149172	0.051*	

### 1-[(6-tert-Butyl-2-oxo-2H-chromen-4-yl)methyl]-4,4-dimethylpiperidine-2,6-dione Atomic displacement parameters (Å^2^)

**Table:**

	*U*^11^	*U*^22^	*U*^33^	*U*^12^	*U*^13^	*U*^23^
O1	0.0257 (6)	0.0192 (5)	0.0227 (6)	−0.0010 (4)	0.0068 (4)	0.0010 (4)
O2	0.0283 (6)	0.0266 (6)	0.0249 (6)	0.0007 (5)	0.0028 (5)	0.0079 (5)
O3	0.0210 (6)	0.0304 (6)	0.0330 (6)	−0.0012 (5)	0.0090 (5)	−0.0052 (5)
O4	0.0452 (8)	0.0524 (8)	0.0468 (8)	−0.0083 (6)	0.0309 (7)	−0.0176 (7)
N1	0.0227 (7)	0.0174 (6)	0.0184 (6)	−0.0005 (5)	0.0053 (5)	−0.0029 (5)
C10	0.0241 (8)	0.0306 (9)	0.0170 (7)	−0.0053 (6)	0.0078 (6)	−0.0037 (6)
C3	0.0174 (7)	0.0251 (8)	0.0217 (8)	0.0000 (6)	0.0060 (6)	0.0003 (6)
C2	0.0191 (7)	0.0222 (8)	0.0196 (7)	−0.0004 (6)	0.0048 (6)	−0.0021 (6)
C1	0.0172 (7)	0.0205 (8)	0.0193 (7)	0.0008 (6)	0.0071 (6)	−0.0019 (6)
C8	0.0209 (7)	0.0203 (7)	0.0196 (8)	−0.0006 (6)	0.0083 (6)	−0.0023 (6)
C7	0.0188 (7)	0.0268 (8)	0.0200 (8)	−0.0036 (6)	0.0101 (6)	−0.0037 (6)
C6	0.0265 (8)	0.0279 (8)	0.0227 (8)	−0.0094 (7)	0.0111 (6)	−0.0096 (7)
C5	0.0294 (8)	0.0195 (7)	0.0284 (8)	−0.0055 (6)	0.0139 (7)	−0.0045 (6)
C4	0.0181 (7)	0.0220 (8)	0.0197 (7)	0.0004 (6)	0.0089 (6)	0.0001 (6)
C9	0.0165 (7)	0.0202 (7)	0.0192 (7)	0.0005 (6)	0.0081 (6)	−0.0027 (6)
C11	0.0780 (16)	0.0566 (14)	0.0200 (9)	0.0206 (12)	0.0053 (9)	−0.0056 (9)
C13	0.0956 (19)	0.0824 (18)	0.0232 (10)	−0.0579 (16)	−0.0085 (11)	0.0168 (11)
C12	0.0478 (12)	0.119 (2)	0.0345 (11)	0.0451 (14)	0.0214 (10)	0.0339 (13)
C14	0.0276 (8)	0.0202 (8)	0.0162 (7)	0.0003 (6)	0.0024 (6)	−0.0016 (6)
C19	0.0217 (8)	0.0159 (7)	0.0221 (8)	0.0034 (6)	0.0067 (6)	0.0023 (6)
C18	0.0248 (8)	0.0224 (8)	0.0196 (8)	0.0006 (6)	0.0075 (6)	−0.0027 (6)
C17	0.0275 (8)	0.0191 (7)	0.0192 (8)	−0.0042 (6)	0.0039 (6)	−0.0008 (6)
C16	0.0223 (8)	0.0257 (8)	0.0278 (8)	−0.0049 (6)	0.0086 (7)	0.0017 (7)
C15	0.0288 (8)	0.0216 (8)	0.0281 (8)	−0.0005 (6)	0.0131 (7)	−0.0001 (6)
C21	0.0381 (10)	0.0325 (9)	0.0251 (9)	−0.0118 (8)	0.0029 (7)	−0.0031 (7)
C20	0.0481 (11)	0.0196 (8)	0.0270 (9)	−0.0041 (7)	0.0065 (8)	−0.0007 (7)

### 1-[(6-tert-Butyl-2-oxo-2H-chromen-4-yl)methyl]-4,4-dimethylpiperidine-2,6-dione Geometric parameters (Å, º)

**Table:**

O1—C3	1.3732 (18)	C11—H11B	0.9600
O1—C4	1.3798 (17)	C11—H11C	0.9600
O2—C3	1.2104 (18)	C13—H13A	0.9600
O3—C19	1.2176 (18)	C13—H13B	0.9600
O4—C15	1.212 (2)	C13—H13C	0.9600
N1—C19	1.396 (2)	C12—H12A	0.9600
N1—C15	1.401 (2)	C12—H12B	0.9600
N1—C14	1.4627 (18)	C12—H12C	0.9600
C10—C12	1.505 (2)	C14—H14A	0.9700
C10—C13	1.513 (3)	C14—H14B	0.9700
C10—C11	1.519 (2)	C19—C18	1.503 (2)
C10—C7	1.534 (2)	C18—C17	1.527 (2)
C3—C2	1.447 (2)	C18—H18A	0.9700
C2—C1	1.345 (2)	C18—H18B	0.9700
C2—H2	0.9300	C17—C16	1.527 (2)
C1—C9	1.456 (2)	C17—C21	1.528 (2)
C1—C14	1.509 (2)	C17—C20	1.530 (2)
C8—C7	1.388 (2)	C16—C15	1.497 (2)
C8—C9	1.404 (2)	C16—H16A	0.9700
C8—H8	0.9300	C16—H16B	0.9700
C7—C6	1.397 (2)	C21—H21A	0.9600
C6—C5	1.383 (2)	C21—H21B	0.9600
C6—H6	0.9300	C21—H21C	0.9600
C5—C4	1.382 (2)	C20—H20A	0.9600
C5—H5	0.9300	C20—H20B	0.9600
C4—C9	1.395 (2)	C20—H20C	0.9600
C11—H11A	0.9600		
			
C3—O1—C4	121.19 (12)	H12A—C12—H12B	109.5
C19—N1—C15	124.25 (12)	C10—C12—H12C	109.5
C19—N1—C14	117.76 (12)	H12A—C12—H12C	109.5
C15—N1—C14	117.99 (12)	H12B—C12—H12C	109.5
C12—C10—C13	110.3 (2)	N1—C14—C1	113.77 (12)
C12—C10—C11	108.92 (17)	N1—C14—H14A	108.8
C13—C10—C11	107.38 (18)	C1—C14—H14A	108.8
C12—C10—C7	107.88 (13)	N1—C14—H14B	108.8
C13—C10—C7	111.17 (13)	C1—C14—H14B	108.8
C11—C10—C7	111.20 (14)	H14A—C14—H14B	107.7
O2—C3—O1	116.87 (14)	O3—C19—N1	119.72 (14)
O2—C3—C2	125.52 (14)	O3—C19—N1	119.72 (14)
O1—C3—C2	117.62 (13)	O3—C19—C18	122.81 (14)
C1—C2—C3	122.49 (14)	O3—C19—C18	122.81 (14)
C1—C2—H2	118.8	N1—C19—C18	117.44 (13)
C3—C2—H2	118.8	C19—C18—C17	114.68 (12)
C2—C1—C9	118.92 (14)	C19—C18—H18A	108.6
C2—C1—C14	122.73 (13)	C17—C18—H18A	108.6
C9—C1—C14	118.35 (12)	C19—C18—H18B	108.6
C7—C8—C9	122.06 (14)	C17—C18—H18B	108.6
C7—C8—H8	119.0	H18A—C18—H18B	107.6
C9—C8—H8	119.0	C18—C17—C16	107.02 (12)
C8—C7—C6	117.65 (14)	C18—C17—C21	109.46 (13)
C8—C7—C10	120.93 (13)	C16—C17—C21	109.73 (13)
C6—C7—C10	121.33 (13)	C18—C17—C20	110.55 (13)
C5—C6—C7	121.83 (14)	C16—C17—C20	110.17 (13)
C5—C6—H6	119.1	C21—C17—C20	109.87 (13)
C7—C6—H6	119.1	C15—C16—C17	114.94 (13)
C4—C5—C6	119.18 (14)	C15—C16—H16A	108.5
C4—C5—H5	120.4	C17—C16—H16A	108.5
C6—C5—H5	120.4	C15—C16—H16B	108.5
O1—C4—C5	116.83 (13)	C17—C16—H16B	108.5
O1—C4—C9	121.83 (13)	H16A—C16—H16B	107.5
C5—C4—C9	121.34 (14)	O4—C15—N1	119.66 (15)
C4—C9—C8	117.90 (13)	O4—C15—N1	119.66 (15)
C4—C9—C1	117.95 (13)	O4—C15—C16	122.71 (15)
C8—C9—C1	124.15 (13)	O4—C15—C16	122.71 (15)
C10—C11—H11A	109.5	N1—C15—C16	117.62 (13)
C10—C11—H11B	109.5	C17—C21—H21A	109.5
H11A—C11—H11B	109.5	C17—C21—H21B	109.5
C10—C11—H11C	109.5	H21A—C21—H21B	109.5
H11A—C11—H11C	109.5	C17—C21—H21C	109.5
H11B—C11—H11C	109.5	H21A—C21—H21C	109.5
C10—C13—H13A	109.5	H21B—C21—H21C	109.5
C10—C13—H13B	109.5	C17—C20—H20A	109.5
H13A—C13—H13B	109.5	C17—C20—H20B	109.5
C10—C13—H13C	109.5	H20A—C20—H20B	109.5
H13A—C13—H13C	109.5	C17—C20—H20C	109.5
H13B—C13—H13C	109.5	H20A—C20—H20C	109.5
C10—C12—H12A	109.5	H20B—C20—H20C	109.5
C10—C12—H12B	109.5		
			
C4—O1—C3—O2	178.97 (13)	C15—N1—C14—C1	−93.71 (16)
C4—O1—C3—C2	−0.63 (19)	C2—C1—C14—N1	−4.4 (2)
O2—C3—C2—C1	−178.90 (14)	C9—C1—C14—N1	175.64 (12)
O1—C3—C2—C1	0.7 (2)	O3—O3—C19—N1	0.00 (3)
C3—C2—C1—C9	−0.2 (2)	O3—O3—C19—C18	0.00 (4)
C3—C2—C1—C14	179.84 (14)	C15—N1—C19—O3	178.33 (14)
C9—C8—C7—C6	−1.5 (2)	C14—N1—C19—O3	−0.9 (2)
C9—C8—C7—C10	−178.19 (13)	C15—N1—C19—O3	178.33 (14)
C12—C10—C7—C8	86.1 (2)	C14—N1—C19—O3	−0.9 (2)
C13—C10—C7—C8	−34.9 (2)	C15—N1—C19—C18	0.1 (2)
C11—C10—C7—C8	−154.54 (16)	C14—N1—C19—C18	−179.08 (12)
C12—C10—C7—C6	−90.5 (2)	O3—C19—C18—C17	153.26 (14)
C13—C10—C7—C6	148.46 (19)	O3—C19—C18—C17	153.26 (14)
C11—C10—C7—C6	28.9 (2)	N1—C19—C18—C17	−28.58 (18)
C8—C7—C6—C5	1.1 (2)	C19—C18—C17—C16	52.45 (16)
C10—C7—C6—C5	177.77 (13)	C19—C18—C17—C21	171.31 (13)
C7—C6—C5—C4	0.3 (2)	C19—C18—C17—C20	−67.54 (17)
C3—O1—C4—C5	179.81 (13)	C18—C17—C16—C15	−51.80 (17)
C3—O1—C4—C9	0.2 (2)	C21—C17—C16—C15	−170.48 (13)
C6—C5—C4—O1	178.96 (13)	C20—C17—C16—C15	68.43 (17)
C6—C5—C4—C9	−1.4 (2)	O4—O4—C15—N1	0.0 (2)
O1—C4—C9—C8	−179.38 (12)	O4—O4—C15—C16	0.00 (13)
C5—C4—C9—C8	1.0 (2)	C19—N1—C15—O4	−178.11 (15)
O1—C4—C9—C1	0.3 (2)	C14—N1—C15—O4	1.1 (2)
C5—C4—C9—C1	−179.32 (13)	C19—N1—C15—O4	−178.11 (15)
C7—C8—C9—C4	0.5 (2)	C14—N1—C15—O4	1.1 (2)
C7—C8—C9—C1	−179.19 (13)	C19—N1—C15—C16	0.6 (2)
C2—C1—C9—C4	−0.3 (2)	C14—N1—C15—C16	179.80 (12)
C14—C1—C9—C4	179.69 (13)	C17—C16—C15—O4	−154.14 (16)
C2—C1—C9—C8	179.38 (14)	C17—C16—C15—O4	−154.14 (16)
C14—C1—C9—C8	−0.6 (2)	C17—C16—C15—N1	27.2 (2)
C19—N1—C14—C1	85.53 (16)		

### 1-[(6-tert-Butyl-2-oxo-2H-chromen-4-yl)methyl]-4,4-dimethylpiperidine-2,6-dione Hydrogen-bond geometry (Å, º)

**Table:**

*D*—H···*A*	*D*—H	H···*A*	*D*···*A*	*D*—H···*A*
C16—H16*A*···O2^i^	0.97	2.41	3.1800 (19)	136
C14—H14*A*···O3^ii^	0.97	2.49	3.3898 (18)	155
C2—H2···O2^i^	0.93	2.50	3.3687 (18)	155
C14—H14*A*···O3	0.97	2.40	2.6915 (19)	97
C14—H14*B*···O4	0.97	2.34	2.698 (2)	101

Symmetry codes: (i) −*x*+1/2, −*y*+3/2, −*z*; (ii) −*x*+1, *y*, −*z*+1/2.

### The energy values of the global reactivity descriptors for the title compound

**Table:**

E_homo *(eV)*	-6.59
E_lumo *(eV)*	-2.06
Energy gap =(E_lumo)-(E_homo) *(eV)*	4.53
Ionisation Energy(I) *(eV)*	6.59
Electron Affinity(A)*(eV)*	2.06
Electronegativity(χ)*(eV)*	4.325
Chemical Hardness(η)*(eV)*	2.265
Chemical Softness(S) *(eV)^-1^*	0.221
Chemical Potential(µ) *(eV)*	-4.325
Electrophilicity Index(ω) *(eV)*	4.129
